# Interfacial engineering of ferromagnetism in wafer-scale van der Waals Fe_4_GeTe_2_ far above room temperature

**DOI:** 10.1038/s41467-023-37917-8

**Published:** 2023-04-29

**Authors:** Hangtian Wang, Haichang Lu, Zongxia Guo, Ang Li, Peichen Wu, Jing Li, Weiran Xie, Zhimei Sun, Peng Li, Héloïse Damas, Anna Maria Friedel, Sylvie Migot, Jaafar Ghanbaja, Luc Moreau, Yannick Fagot-Revurat, Sébastien Petit-Watelot, Thomas Hauet, John Robertson, Stéphane Mangin, Weisheng Zhao, Tianxiao Nie

**Affiliations:** 1 Fert Beijing Institute, MIIT Key Laboratory of Spintronics, School of Integrated Circuit Science and Engineering, Beihang University, Beijing, 100191 China; 2 Universite de Lorraine, Institut Jean Lamour, UMR CNRS 7198 Nancy, France; 3 Engineering Department, Cambridge University, Cambridge, CB2 1PZ UK; 4 School of Materials Science and Engineering, Beihang University, Beijing, 100191 China; 5 Department of Electrical and Computer Engineering, Auburn University, Auburn, AL USA; 6 Fachbereich Physik and Landesforschungszentrum OPTIMAS, Technische Universität Kaiserslautern, 67663 Kaiserslautern, Germany

**Keywords:** Two-dimensional materials, Magnetic properties and materials

## Abstract

Despite recent advances in exfoliated vdW ferromagnets, the widespread application of 2D magnetism requires a Curie temperature (T_c_) above room temperature as well as a stable and controllable magnetic anisotropy. Here we demonstrate a large-scale iron-based vdW material Fe_4_GeTe_2_ with the T_c_ reaching ~530 K. We confirmed the high-temperature ferromagnetism by multiple characterizations. Theoretical calculations suggested that the interface-induced right shift of the localized states for unpaired Fe *d* electrons is the reason for the enhanced T_c_, which was confirmed by ultraviolet photoelectron spectroscopy. Moreover, by precisely tailoring Fe concentration we achieved arbitrary control of magnetic anisotropy between out-of-plane and in-plane without inducing any phase disorders. Our finding sheds light on the high potential of Fe_4_GeTe_2_ in spintronics, which may open opportunities for room-temperature application of all-vdW spintronic devices.

## Abstract (web summary)

Applications of van der Waals magnetic systems are typically hampered by the low Curie temperature of van der Waals magnets. Here, Wang et al use molecular beam epitaxy to grow large films of Fe_4_GeTe_2_ with Curie temperatures over 500 K, and the film’s magnetic anisotropy can be tuned arbitrarily by controlling stoichiometry.

## Introduction

Replacing bulk materials with two-dimensional (2D) materials is regarded as a promising way to continue scaling electronic devices as Moore’s law is claimed to meet a dead end and the device scaling approaches quantum limit^1^. With the van der Waals (vdW) layered structures, 2D materials present a unique opportunity potentially outperforming conventional bulk materials due to their reduced dimensions which massively suppress interfacial bonds, and eliminate gap states. As an emerging member of the vdW family, 2D ferromagnetic materials that use the electron spins as another degree of freedom could pave the way for high-performance, energy-efficient, and non-volatile vdW spintronic devices beyond Moore’s law^2,3^. To meet device operating standards in industry or aerospace, such a device would need to withstand operating temperatures exceeding 125 °C (~400 K)^4^. Current Curie temperatures (T_c_) of 2D ferromagnetic materials are far below this, and there is an urgent need for new materials with high-temperature ferromagnetic order above 400 K amenable large-scale production.

For 2D magnets, magnetic anisotropy plays an important role in sustaining the long-range ferromagnetic order at a finite temperature^5,6^. Strong magnetic anisotropy is also a key requirement for satisfying different functionalities in spintronic devices. For instance, materials with perpendicular magnetic anisotropy (PMA) demonstrate a lower switching current and better scalability than materials with in-plane magnetic anisotropy (IMA) for the configuration of magnetic random-access memory. In contrast, an IMA pinned layer may be needed to combine with a PMA-free layer for higher sensitivity in magnetic sensors^7^. For conventional magnets, the magnetic anisotropy can be controlled by engineering the interface. However, it is quite challenging to manipulate the magnetic anisotropy of 2D ferromagnetic materials, especially at room temperature. Finding a 2D ferromagnetic material with controllable anisotropy is urgent for developing 2D vdW spintronic devices in the future.

Recently, the emergence of Fe-rich vdW ferromagnets Fe_n_GeTe_2_ received intense research interest due to their large magnetization near room temperature (for 3 ≤ *n* ≤ 5, T_c_ = 220–310 K)^8–15^. In the family of Fe_n_GeTe_2_, Fe_4_GeTe_2_ has been highlighted as a very promising candidate material for spintronics applications because of its relatively high T_c_ (270 K) and flexible magnetic anisotropy^10,16–18^. In traditional vdW ferromagnetic materials (for instance, CrI_3_ or Cr_2_Ge_2_Te_6_), the magnetic atoms are encapsulated by nonmagnetic atoms and form a 2D magnetic system^19,20^. The pair-exchange interaction between the neighboring magnetic atoms in this 2D plane is weak, resulting in a low T_c_. In Fe_4_GeTe_2_, however, the Fe-Fe dumbbells form a corrugated honeycomb lattice, and this 3D arrangement of magnetic atoms enhances the spin interaction as well as the T_c_ in Fe_4_GeTe_2_. Moreover, the magnetic anisotropy of Fe_4_GeTe_2_ is temperature-dependent^17,18^, and exhibits an anisotropy transition from IMA to PMA as the temperature decreases because of the competition between the magnetocrystalline anisotropy and the shape anisotropy^21^. However, realistic applications require a substantial increase in its T_c_ and arbitrary control of its magnetic anisotropy. Fortunately, pioneering works have shown that the magnetism of vdW materials can be engineered by external stimuli, such as gating^22–25^, pressure and strain^26–28^, laser^29^, and proximity effect^30,31^. These methods may offer an avenue for simultaneously harnessing the T_c_ and anisotropy in Fe_4_GeTe_2_ to satisfy industry standards for future 2D vdW spintronic applications.

In this work, we successfully synthesized wafer-scale vdW Fe_4_GeTe_2_ with controlled thickness by molecular beam epitaxy (MBE). We found that the Tc of the Fe_4_GeTe_2_ thin film can be precisely tailored by tuning the thickness and Fe concentration and the value was enhanced to over 500 K, which is high enough to meet the demands of most spintronic devices for high working temperature and is compatible with multiple modern patterning processes. Theoretical analysis revealed that the high-temperature ferromagnetism arises from interfacial orbital coupling, which moves the localized states of the Fe_4_GeTe_2_ toward the Fermi level, leading to an increase of the exchange interaction as well as T_c_. Meanwhile, we also obtained a stable and controllable magnetic anisotropy in our Fe_4_GeTe_2_ through modulating Fe concentration. As it increases, the Fe_4_GeTe_2_ demonstrates an anisotropy transition from PMA to IMA without introducing any phase disorders, exhibiting its great potential as a universal base material for magnetic memory or magnetic sensor devices. Our result provides a way for realizing high-T_c_ and tunable magnetic anisotropy in vdW material.

## Results

**Fig. 1:**
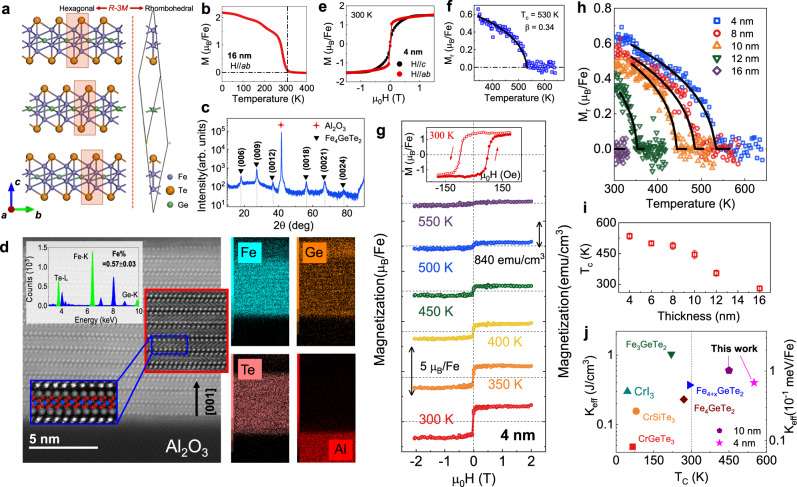
Crystal structure of the Fe_4_GeTe_2_ and its thickness-dependent magnetism. **a** Schematics of the crystal structure of Fe_4_GeTe_2_ stacked in ABC configuration (left), and its rhombohedral structure unit (right). **b** Temperature dependence of the magnetization for 16 nm Fe_4_GeTe_2_. **c** XRD scan of the Fe_4_GeTe_2_ film, showing Al_2_O_3_ {001} and Fe_4_GeTe_2_ {003} family peaks. **d** A typical HRTEM image of Fe_4_GeTe_2_ films, the color squares show the high-pass filtered images of the vdW structure. Up Inset: The EDX result verifies the 4:1:2 Fe:Ge:Te stoichiometric composition with the uniform element distribution map (right). **e** Room-temperature magnetic hysteresis loops of 4 nm Fe_4_GeTe_2_, the magnetism can survive at an extremely high temperature, the T_c_ is estimated to be 530 K from the power-law fitting of the in-plane M_r_–T curve in (**f**). **g** Detailed magnetic field-dependent magnetization of 4 nm Fe_4_GeTe_2_ at various temperatures for H//*ab*. Inset: zoom-in hysteresis loop at 300 K. **h** Temperature-dependent M_r_ for Fe_4_GeTe_2_ with the thickness of 4, 8, 10, 12, and 16 nm. **i** T_c_ for Fe_4_GeTe_2_ thin films with different thicknesses, a negative correlation can be observed between the thickness and the T_c_ of the films. The error bars describe the deviation during the power-law fitting. **j** Effective magnetic anisotropy energy *K*_*eff*_ and Curie temperature T_c_ for our samples and previous vdW ferromagnets^18,34–38^.

The Fe_4_GeTe_2_ has a rhombohedral crystal structure with an R-3*m* space group, as shown in Fig. 1a. Similar to Fe_3_GeTe_2_ (Fig. S1a), Fe_4_GeTe_2_ has the structural units of Fe-Fe dumbbells, which are alternatingly off from the horizontal line and form a corrugated honeycomb lattice hosting one Ge atom at the center. In each Fe_4_GeTe_2_ monolayer (ML), the covalently-bonded Fe4Ge heterometallic slab is sandwiched between two Te layers. The thickness of an ML is ~9.7 Å and adjacent MLs are separated by a vdW gap. Previous works on the exfoliated bulk Fe_4_GeTe_2_ have indicated that the T_c_ of Fe_4_GeTe_2_ can be high because of the relatively strong spin-exchange interaction between the neighboring magnetic atoms^15,18^. In this work, the 16 nm Fe_4_GeTe_2_ also demonstrates a near room temperature T_c_, which can be proved by the in-plane M-T curve in Fig. 1b. Here we extract its saturation magnetization (M_s_) of 2.13 μ_B_/Fe, which is higher than 1.6 μ_B_/Fe in Fe_3_GeTe_2_ single crystal^8^. To elucidate its crystal structure, X-ray diffraction (XRD) measurement was performed and a typical spectrum is shown in Fig. 1c. The peaks are well ascribed to the {003} family planes of Fe_4_GeTe_2_, featuring a perfect single crystal^17^. The vdW stacking structure is further demonstrated by the high-resolution transmission electron microscopy (HRTEM) image in Fig. 1d. The staggered Te monolayer distinguishes the structure of Fe_4_GeTe_2_ from that of Fe_3_GeTe_2_, which also leads to a different structural characteristic in the XRD spectrum (as shown in Fig. S1b). Chemical analysis using energy-dispersive X-ray spectroscopy (EDX) confirms the stoichiometry of Fe_4_GeTe_2_ in Fig. S2, and EDX mapping (the right side of Fig. 1d) shows that all the elements of Fe, Ge, and Te are uniformly distributed in the film. Together, these results prove that a high-quality single-crystalline Fe_4_GeTe_2_ vdW film was synthesized by MBE in our work.

Traditionally vdW ferromagnets suffer a sharp decrease in T_c_ when the thickness decreases to the atomic level. It is because that the exchange interactions alone are not able to sustain the magnetic order at finite temperature due to the thermal fluctuations in two dimensions^23^. However, the T_c_ shows a distinguishing thickness-dependent feature in our samples. Here, a 4 nm Fe_4_GeTe_2_ was prepared, and the ferromagnetism was demonstrated by multiple magnetic characterizations, including vibrating sample magnetometer (VSM), magneto-optical Kerr effect (MOKE), ferromagnetic resonance (FMR), and magnetotransport measurements (Fig. S3). Figure 1e shows the M-H loops taken along the *ab* and *c* axis and a robust IMA can be observed even at 300 K. The T_c_ of the sample can be deduced precisely from the in-plane remanent magnetization (M_r_), as shown in Fig. 1f, which shows a power-law dependence on the temperature as ${{{{{{\rm{M}}}}}}}_{{{{{{\rm{r}}}}}}}({{{{{\rm{T}}}}}})\propto {({{{{{{\rm{T}}}}}}}_{{{{{{\rm{c}}}}}}}-{{{{{\rm{T}}}}}})}^{{{{{{\rm{\beta }}}}}}}$. Here the T_c_ = 530 K, which is much higher than that of previous iron-based vdW ferromagnets^14,15,18,32^, and the value of β is estimated to be 0.34, which follows the case of the quasi-2D Heisenberg model in easy axis^6,33^. To further illustrate the magnetic properties, high-temperature M-H loops are displayed in Fig. 1g under an in-plane magnetic field. The inset shows the zoom-in hysteresis at 300 K, with clearly resolvable coercivity verifying the high-temperature magnetism of the sample. A hysteresis loop can still be seen even at 500 K, indicating the presence of high-temperature magnetism in the few-layer Fe_4_GeTe_2_.

To precisely depict the thickness dependence of T_c_, M_r_–T curves for samples of different thicknesses are plotted in Fig. 1h. All M_r_–T curves can be fitted with the Heisenberg model, and the relation between the T_c_ and thickness is summarized in Fig. 1i for more clarity. For 4 nm Fe_4_GeTe_2_, magnetic order survives up to ~530 K, while the T_c_ decreases to ~270 K as the thickness reaches 16 nm. This trend illustrates the interface modulation on magnetism in Fe_4_GeTe_2_, the bottom few layers are expected to be affected most by the interface, thereby presenting the highest T_c_. As an attempt to confirm the interface-enhanced T_c_, we prepared a mechanical exfoliated Fe_4_GeTe_2_ flake, which was cleaved from a 10 nm epitaxial Fe_4_GeTe_2_ film, and measured its magnetic properties by MOKE (Fig. S4). After exfoliation from the substrate, the Fe_4_GeTe_2_ flake cannot sustain room-temperature magnetism. This serves as a strong indication that the T_c_ enhancement comes from the interfacial modulation between the Fe_4_GeTe_2_ film and the sapphire substrate. Figure 1j shows a comparison of the magnetic properties between our samples and previous vdW ferromagnets^18,34–38^. Our Fe_4_GeTe_2_ presents the highest T_c_ among all the materials while keeping its relatively high magnetic anisotropy.

**Fig. 2:**
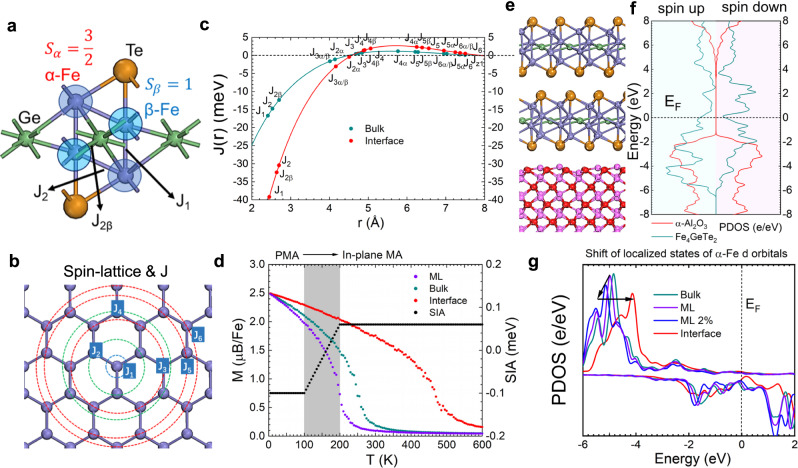
First-principle calculation of the magnetic properties of Fe_4_GeTe_2._ **a** Atomic structure of the unit cell of Fe_4_GeTe_2_. Two kinds of Fe with different symmetric positions and spins are marked as α-Fe and β-Fe. The most important three exchange interactions are marked as *J*_*1*_*, J*_*2*_, and *J*_*2β*_. **b** Top view of the spin-lattice of Fe_4_GeTe_2_ and marked exchange interactions, only Fe atoms are shown. **c** *J-r* dispersion of bulk and interface-modulated Fe_4_GeTe_2_ obeying the RKKY rule. **d** Average magnetization per Fe versus temperature for the ML, bulk, and interface-modulated Fe_4_GeTe_2_. The SIA is marked in black. **e** Atomic structure of 2-layers Fe_4_GeTe_2_ onto the sapphire substrate. **f** PDOS of the Fe_4_GeTe_2_ and the substrate. **g** PDOS of the *d* orbitals of the α-Fe in bulk, ML with 2% strain and interface-modulated Fe_4_GeTe_2_.

In the following, density functional theory (DFT) calculations were performed to unveil the mechanism beyond the T_c_ enhancement. First, electronic structure and the internal exchange interactions of Fe_4_GeTe_2_ were determined by ab inito calculation (details in the Supplementary Materials Figs. S5 and S6). As shown in Fig. 2a, Fe_4_GeTe_2_ has two different Fe sites, namely as α-Fe and β-Fe. The α-Fe is more localized and has a larger spin than the itinerant β-Fe. Our calculation reveals that the spin at the α-Fe is 3/2 while that of the β-Fe is 1 in both the monolayer and bulk form. Therefore, the average magnetic moment per Fe near 0 K is 2.5 μ_B_, which is consistent with our experimental result. The calculated primitive cell structure of single-layer Fe_4_GeTe_2_ contains a dumbbell shape of four Fe atoms, as shown in Fig. 2a. There are three nearest-neighbor exchange interactions called *J*_*1*_, *J*_*2*_, and *J*_*2β*_, at a spatial distance of ~2.5 Å. The other distant exchange interactions are more than 4 Å and are less important. The naming rule of the exchange interactions is illustrated in Fig. 2b (also in the Supplementary Materials Fig. S6). Although the itinerant magnetism in Fe_4_GeTe_2_ should be described by the Stoner model^39^, the local magnetic moments of Fe are large enough that such itinerant ferromagnetism can be equivalently transformed into the classic Heisenberg model with Ruderman–Kittle–Kasuya–Yosida (RKKY) exchange^40^. The exchange interactions follow the RKKY rule^41^:

1 $$J(r)=-{J}^{2}\times \frac{{{{{{\rm{sin}}}}}}(2{k}_{F}r)-2{k}_{F}r{{{{{\rm{cos}}}}}}(2{k}_{F}r)}{{({k}_{F}r)}^{4}}$$

where *r* is the distance between the two spin sites and *k*_*F*_ is the Fermi momentum of the itinerant electrons. Kondo model treats localized electrons as magnetic impurities embedded in the itinerant conducting electrons and *J* describes the exchange interaction of a localized spin coupled to delocalized electrons. The RKKY model further describes the interaction between two localized spins via the conduction electrons. Figure 2c shows *J*(*r*) relations from the nearest exchanges to far above 8 Å.

To further simulate T_c_, Metropolis Monte-Carlo (MMC) method was adopted to calculate the temperature-dependent magnetic moments per Fe. The Hamiltonian can be written as:

2 $$H=\mathop{\sum}\limits_{i < j}{s}_{i}^{\alpha }{[\,{J}_{ij}]}_{\alpha \beta }{s}_{j}^{\beta }+\mathop{\sum}\limits_{i}A{({s}_{i}^{z})}^{2}$$

where *s*_*i*_^*α*^ is the spin operator on site *i*, and *α, β* can be *x, y, z*. [ *J*_*ij*_]_*αβ*_ describes the Heisenberg exchange interactions between the spins on site *i* and *j*, which is a 3 by 3 matrix. Considering the centrosymmetric structure of Fe_4_GeTe_2_, we neglect the non-isotropic exchanges like in Fe_3_GeTe_2_ or chromium-based compound^23,31^, the Heisenberg exchange then degenerates into a scalar. The coefficient of A is the single-ion perpendicular magnetocrystalline anisotropy energy (SIA), which stems from spin-orbit coupling. With the SIA, a ferromagnetic order can be established in 2D at finite temperatures via breaking the continuous rotational symmetry of the Hamiltonian and rendering a gap in the ground state magnon mode. Considering the transition of SIA, the MMC calculation in Fig. 2d predicts the T_c_ of intrinsic Fe_4_GeTe_2_ monolayer is ~200 K while the value of bulk form is ~270 K. Notably, the calculated bulk T_c_ (cyan line) is consistent with the bulk value of previous exfoliated sample^17,18^ as well as our experimental result in Fig. 1h. For the monolayer, however, the calculated value (purple line) is close to the exfoliated sample but far below the T_c_ measured in our Fe_4_GeTe_2_. This result indicates the accuracy of our calculation and confirms again the interfacial modulation in our Fe_4_GeTe_2_/Al_2_O_3_ heterostructure.

Here we establish a model to simulate the heterostructure and quantify the interface contribution to the T_c_ enhancement. Considering the ~20% lattice difference between the sapphire substrate (α-Al_2_O_3_) and Fe_4_GeTe_2_, the elastic energy is too large compared to the adsorption energy for a 1:1 lattice-matched interface of Fe_4_GeTe_2_ and α-Al_2_O_3_ (Fig. S7). We infer that Fe_4_GeTe_2_ rotates 30 degrees and adsorbs onto the substrate with a tensile strain of ~2% during the epitaxy. This lattice match scheme can be described as ${{{{{{\rm{Fe}}}}}}}_{4}{{{{{{\rm{GeTe}}}}}}}_{2}{:{{{{{\rm{Al}}}}}}}_{2}{{{{{{\rm{O}}}}}}}_{3}=2:\sqrt{3}$, which was verified by the RHEED patterns, as shown in Fig. S8. Figure 2e shows the side view of the interface, and the partial density of states (PDOS) of both Fe_4_GeTe_2_ and Al_2_O_3_ are demonstrated in Fig. 2f. Here the *E*_*F*_ lies near the valence edge of Al_2_O_3_, and the bandgap of Al_2_O_3_ is calculated to be closed to 6 eV. The spin-down bands of the metal inside the gap are mostly unoccupied while that of spin-up are occupied.

According to the mean-field theory, T_c_ is proportional to the overall exchange interaction as well as S(S + 1), where S is the average onsite spin on Fe and the value is determined by the density difference between up and down states. Notably, in our samples, the spin does not change at the interface because the *E*_*F*_ lies between the spin up and spin down *d* bands and is quite far from either of them (Figs. S5b and S7d). Therefore, the enhancement of the exchange interactions is the main reason for the dramatic T_c_ increase in Fig. 2d. The T_c_ of the interfacial Fe_4_GeTe_2_ is predicted to be nearly 500 K, much higher than its ML and bulk forms without interfacial contact, which is consistent with our experimental observation. Notably, the change of the exchange interactions stems from *J* rather than the Fe-Fe distances *r* as it cannot be changed a lot (Fig. 2c and Fig. S7b). According to the Anderson model, *J* is related to the distance between the Fermi level (*E*_*F*_) and the localized states that contribute to the formation of magnetic moments (*E*_*d*_), as well as the Hubbard U which denotes the onsite Coulomb repulsion^42,43^:

3 $$J \sim -\frac{1}{{E}_{F}-{E}_{d}}+\frac{1}{{E}_{F}-{E}_{d}-U}$$

This is under the condition that *E*_*d*_ < *E*_*F*_ < *E*_*d*_ + *U*, where the magnetic moment arises from single occupation of the localized level rather than zero or two. The *J*^2^ has a local minimum when the *E*_*F*_ is in the mid of *E*_*d*_ and *E*_*d*_ + *U*. In Fe_4_GeTe_2_, the *E*_*d*_ is the energy level of the unpaired *d* electrons in Fe (Supplementary Materials, Section 2).

As Fig. S9 shows, by contacting with the sapphire substrate, the *d* electrons shift rightwards, while the deeper level *s* and *p* electrons remain almost unchanged, indicating that there is no *E*_*F*_ shifting by the interfacial engineering. Here we calculate the PDOS of α-Fe *d* electrons to inspect the interface-induced *E*_*d*_ shift in Fe_4_GeTe_2_. As shown in Fig. S9d and Fig. 2g (the red curve), compared with the bulk or monolayer forms, the integration with the sapphire induces a right shift of unpaired Fe *d* electrons, which will move the *E*_*d*_ towards the *E*_*F*_, and is the key factor leading to the enhancement of *J*. To verify our theoretical prediction, we used ultraviolet photoelectron spectroscopy (UPS) to probe the *E*_*d*_ shift induced by the interface in our Fe_4_GeTe_2_ thin film, as shown in Fig. S10. As the thickness decreases, we have observed states accumulated towards the *E*_*F*_, which comes from the right shift of the Fe *d* localized states. The experimental results are consistent with our theoretical calculation in Fig. 2g. According to the Anderson model, the right shift of *E*_*d*_ results in the enhancement of *J* as well as the T_c_, which is the direct consequence of sapphire-induced interfacial orbital coupling. Considering the slight extension on the Fe_4_GeTe_2_ lattice with thickness decrease (Fig. S8), we also simulate the *d* orbital PDOS for α-Fe in ML Fe_4_GeTe_2_ with 2% tensile strain (blue line in Fig. 2g). The strain is expected to extend the distance between *E*_*d*_ and *E*_*F*_, which contradicts our UPS results and heterostructure calculation. Therefore, strain is excluded from the factors causing the T_c_ enhancement in Fig. 1h.

**Fig. 3:**
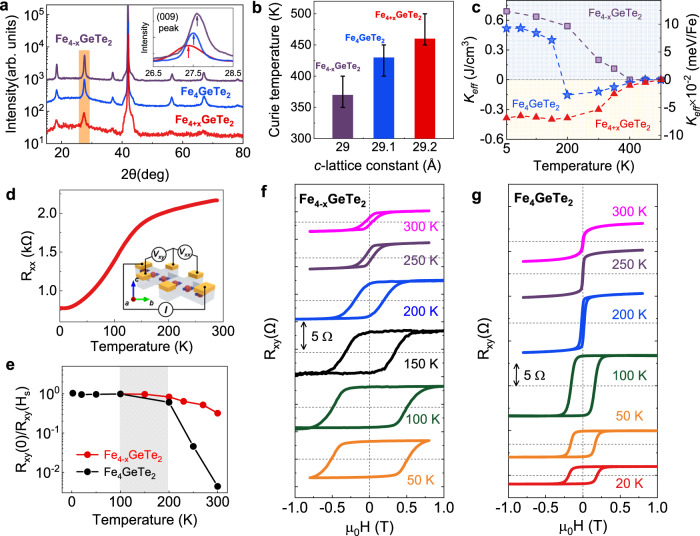
Doping-modulated magnetism in Fe_4_GeTe_2_. **a** XRD scan of the samples with different Fe doping concentrations, which all belong to vdW Fe_4_GeTe_2_ structure without any phase disorders. Inset: the (009) peaks of the samples, reflecting a slight shift of the XRD curves under different Fe concentrations. **b** The *c*-lattice constants and T_c_ of all the samples, both parameters have a positive correlation with the Fe concentration. The error bars describe the deviation in calculated T_c_ between Arrott plots and mean-field fitting (Fig. S11). **c** Effective magnetic anisotropies *K*_*eff*_ as a function of temperature for the samples, an easy axis rotation can be observed in Fe_4_GeTe_2_. **d** Temperature dependence of the longitudinal resistance for 7 nm Fe_4_GeTe_2_ thin film. Inset: the schematic image of the Hall bar device. **e** Normalized Remnant anomalous Hall resistance R_xy_ as a function of temperature, the resistance values are extracted from the anomalous Hall curves of Fe_4-x_GeTe_2_ (**f**) and Fe_4_GeTe_2_ (**g**). With the temperature increase, an anisotropy transition from PMA to IMA can be well observed In Fe_4_GeTe_2_.

Besides high-temperature magnetism, robust and controllable magnetic anisotropy is also essential for the widespread vdW spintronic applications. Recent works have indicated that as the temperature increases, a magnetic anisotropy reorientation from out-of-plane to in-plane direction appears in Fe_4_GeTe_2_^17,18^. Here we attempted to control the magnetic anisotropy with precise manipulation of the stoichiometry in Fe_4_GeTe_2_. Three thin films with the same thickness (10 nm) but different Fe concentrations were prepared (named Fe_4-x_GeTe_2_, Fe_4_GeTe_2_, and Fe_4+x_GeTe_2_). All the samples present the same crystalline phase, proving the high quality, as shown in the XRD results in Fig. 3a. Careful examination finds that the (009) peaks of the samples demonstrate a small shift, which indicates that the Fe concentration increase can lead to a slight enlargement of the *c* lattice constant in Fe_4_GeTe_2_ because more Fe atoms are introduced into the crystal unit^15^. In addition, the enhanced Fe network consists of more spin-pair interactions, which will increase *J* as well as the T_c_ of Fe_4_GeTe_2_, as shown in Fig. 3b. This stoichiometry-dependent T_c_ can be proved by multiple magnetic characterizations (Figs. S11–S14), and has been widely recognized in the previous research^12–15,17,18,44^.

Interestingly, we found that the magnetic anisotropy along either out-of-plane or in-plane can be precisely controlled by tailoring the Fe concentration. To quantitively illustrate the magnetism evolution in all three samples, Fig. 3c depicts the effective anisotropy energy (*K*_*eff*_) values extracted from the M-H loops of the samples (Figs. S11, S12). Here the sign of *K*_*eff*_ demonstrates the magnetic anisotropy along in-plane (negative sign) or out-of-plane (positive sign). Different from Fe_4-x_GeTe_2_ or Fe_4+x_GeTe_2_ which always present a definite direction of magnetic anisotropy at all temperatures, Fe_4_GeTe_2_ presents a temperature-dependent anisotropy from PMA to IMA, which is consistent with the previous report^18^. The value of *K*_*eff*_ is determined by two competing energies, the easy-axis magnetocrystalline anisotropy (*K*_*m*_) and the easy-plane shape anisotropy (*K*_*sh*_), they usually follow a relation as${K}_{eff}={K}_{m}+{K}_{sh}={K}_{m}-({\mu }_{0}/2){M}_{S}^{2}$. Due to the temperature dependence of magnetization, the magnetic anisotropy in Fe_4_GeTe_2_ is expected to demonstrate a temperature-driven transition. Notably, when the Fe concentration increases from Fe_4-x_GeTe_2_ to Fe_4+x_GeTe_2_, the magnetic anisotropy turns from PMA to IMA, which may originate from the *K*_*m*_ variation during the stoichiometry change. Similar to the Fe_3_GeTe_2_ in the previous reports^9,37^, our Fe_4-x_GeTe_2_ demonstrates a strong PMA with a large *K*_*m*_ over 1 J/cm^2^ at 20 K (Fig. S11). However, this value decreases to 0.88 J/cm^2^ in Fe_4_GeTe_2_ and rapidly drops to 0.23 J/cm^2^ for Fe_4+x_GeTe_2_. While in Fe_4+x_GeTe_2_, a robust and stable IMA can be obtained at all temperatures. Thus, we propose that the degradation of the *K*_*m*_ comes from the incline of magnetocrystalline anisotropy during the Fe concentration increase, which may change the crystalline structure and promote the magnetocrystalline anisotropy tilt along the in-plane direction.

The spin reorientation could also be revealed by the magnetotransport measurements and the inset of Fig. 3d depicts the schematic image of the Hall bar device in the measurements. Figure 3d shows a resistance versus temperature (R–T) curve for the 7 nm Fe_4_GeTe_2_, which exhibits a typical metallic behavior. Figure 3e compares the normalized remnant anomalous Hall resistance R_xy_(0)/R_xy_(H_s_) versus the temperatures for Fe_4-x_GeTe_2_ and Fe_4_GeTe_2_, where the R_xy_(0) and the R_xy_(H_s_) are defined as the anomalous Hall resistance at zero and out-of-plane saturated magnetic field, and the values were extracted from the Hall curves in Figs. 3f and 3g. At all temperature ranges, the Fe_4-x_GeTe_2_ always demonstrates a robust value of ~1, indicating the relatively high remanent magnetization due to the strong PMA. For Fe_4_GeTe_2_, despite the value of R_xy_(0)/R_xy_(H_s_) close to 1 at low temperatures, it decreases substantially from 100 K and finally vanishes when the temperature reaches 250 K. The decrease in the remanent magnetization results from the spin reorientation in the Fe_4_GeTe_2_, which gives rise to the IMA while suppressing the PMA term in the magnetism. Figures 3f and 3g show the detailed anomalous Hall curves for 7 nm Fe_4-x_GeTe_2_ and Fe_4_GeTe_2_ films, respectively. A series of square Hall curves can be observed in Fe_4-x_GeTe_2_ with a clear coercivity, even at high temperatures. By contrast, for Fe_4_GeTe_2_, the coercivity disappears when the temperature increases because of the temperature-driven spin reorientation. The evolution from PMA to IMA in Fe_4_GeTe_2_ can be further proved by the Hall curves for H//*ab* (Fig. S15), which show a clear transition from PMA to IMA, consistent with the above results.

In summary, wafer-scale vdW Fe_4_GeTe_2_ was successfully grown by molecular beam epitaxy and exhibited a strong magnetism with a T_c_ over 500 K. A series of thickness-dependent measurements revealed that the enhanced T_c_ is induced by the interface between Fe_4_GeTe_2_ and the substrate. Theoretical analysis proved that the right shift of the unpaired Fe *d* localized states is the key factor for the increase in T_c_. In addition, the magnetic anisotropy of the Fe_4_GeTe_2_ can be flexibly controlled from out-of-plane to in-plane via tuning Fe composition. In low Fe concentration, the perpendicular magnetocrystalline anisotropy dominates the effective magnetic anisotropy while its contribution is overwhelmed by the in-plane shape anisotropy in high Fe concentration. Our work highlights the great potential of van der Waals Fe_4_GeTe_2_ as a platform for further research and implementation of 2D vdW spintronic devices.

## Methods

### Single crystal growth

High-quality Fe_4_GeTe_2_ thin films were grown on a commercial sapphire substrate in the MBE system. Before the growth, the substrate was annealed at 600 °C for an hour to remove the impurity layer and then cooled down to 300 °C. Elemental Fe, Ge, and Te solid sources were evaporated from standard Knudsen cells. The Fe concentration can be manipulated easily by controlling the flux rate of the Fe source for growing Fe_4-x_GeTe_2_, Fe_4_GeTe_2_, and Fe_4+x_GeTe_2_ samples. The deposition was in situ monitored by reflection high-energy electron diffraction (RHEED). After growth, 2 nm Ge was deposited on the films as the protection layer.

### X-ray diffraction (XRD)

After the growth, the sample was characterized by the PANalytical X’Pert PRO MRD diffractometer with Cu Kα radiation. The generator voltage and current of the setup were set to be 45 kV and 40 mA, respectively. The scanning range was 10°–90° with a step size of 0.03° and a time of 20 s/step.

### Superconducting quantum interference device (SQUID)

The Quantum Design MPMS3 SQUID was employed to measure the low-temperature magnetization behavior of our samples (0–300 K) following standard procedures. During the measurements, the magnetic field sweeps were made in no-overshoot, persistent mode, and the temperature sweeps were made at a rate of 3 K per minute. For M_r_–T curves (Fig. 1b), the films were firstly field-cooled from 300 K to 20 K with an in-plane field of 2 T, then the field was set to zero and the remanent magnetization was measured as the temperature increased. Each estimated magnetic moment was determined from an average of two scans.

### High-resolution transmission electron microscopy (HRTEM)

A JEOL-ARM 200 F Cold FEG transmission electron microscope (TEM) was used to investigate the samples’ structure. The operating voltage of the setup is 200 kV with a point resolution of 0.12 nm. A Carl Zeiss NVision 40 SEM/EDS workstation was used to analyze the chemical composition by plotting the energy-dispersive X-ray spectroscopy (EDX) mapping, which collected the Fe K, Ge K, Te L, O K, and Al K edges.

### Vibrating sample magnetometer (VSM)

A commercial Microsense VSM was used to characterize the high-temperature (above 300 K) magnetic features of our samples. Due to the in-plane magnetic anisotropy above room temperature for our Fe_4_GeTe_2_, only in-plane M-H loops were measured by VSM in Fig. 1g, where the hysteresis was obtained for every 50 K and the magnetic field was scanned between −2 T and 2 T with a step of 500 Oe. The background magnetic contribution of the sapphire substrate and the sample holder was subtracted from the raw data. To test the remanent magnetization of the samples (Fig. 1h), the magnetic field was first raised to 2 T along the easy axis to make the samples saturated, then decreased to 0 T. After that, the temperature was increased to 300 K from 700 K at a rate of 5 K/min under zero field. During the heating process, the magnetic moment of the samples is continuously measured by VSM.

### Transport measurement

Hall bar devices with dimensions of 200 μm × 30 μm were fabricated using standard photolithography for the transport measurements. The in-plane and out-of-plane Hall resistances were measured in the standard four-probe configuration using a Quantum Design Physical Property Measurement System (PPMS-7T). During all the transport measurements, the applied DC current is 50 μA.

### Theoretical calculation

Atomic structure, electronic structure calculations as well as magnetic configurations of ferromagnetic order and anti-ferromagnetic order, are implemented by the density functional theory (DFT) code Cambridge Serial Total Energy Package (CASTEP)^45^. Ultrasoft pseudopotential is used with a plane-wave cutoff energy of 340 eV. The Tkatchenko-Scheffler (TS) scheme^46^ of the van der Waals correction is taken into account in the bulk form and the heterostructure. Self-consistent dipole correction is applied in the heterostructure calculation to overcome the periodical error induced by the charge transfer. All structures are relaxed and the residual force is less than 0.01 eV/Å. The exchange-correlation functional is the semi-local generalized gradient approximation (GGA) form of Perdew-Burke-Erzerhof (PBE)^47^. We applied a Hubbard U = 3.5 eV correction only to the *3d* orbitals of the α-Fe. With the exchange interactions and the single-ion anisotropy (SIA) obtained from the previous steps, we conduct the Metropolis Monte Carlo (MMC) calculation based on the Hamiltonian in Eq. (2). The size of the spin-lattice is set 24 × 24 × 8 for bulk and 40 × 40 × 1 for monolayer to capture the paramagnetism above T_c_ and to isolate the mirror images. Each temperature is simulated with 10^5^ loops to converge to the thermal equilibrium state.

## Supplementary information

Supplementary Information

## Data Availability

The data that support the findings of this study are provided in the paper and Supplementary Materials file. Additional data related to this study are available from the corresponding author upon reasonable request.
