# Supplementary material for: Interfacial engineering of ferromagnetism in wafer-scale van der Waals Fe4GeTe2 far above room temperature
Source: Nat Commun. 2023 Apr 29;14:2483. doi: 10.1038/s41467-023-37917-8 (PMC10148834; doi:10.1038/s41467-023-37917-8)
Supplement: Supplementary file 1 — Supplementary Information [file 41467_2023_37917_MOESM1_ESM.pdf]

Supplementary Materials for

Interfacial engineering of ferromagnetism in wafer-scale van der Waals Fe_4GeTe_2 far above room temperature

Hangtian Wang[$1,2], Haichang Lu[$*1,3], Zongxia Guo[1,2], Ang Li[1], Peichen Wu[1], Jing Li[1], Weiran Xie[1], Zhimei Sun[4], Peng Li[5], Héloïse Damas[2], Anna Maria Friedel[2], Sylvie Migot[2], Jaafar Ghanbaja[2], Luc Moreau[2], Yannick Fagot-Revurat[2], Sébastien Petit-Watelot[2], Thomas Hauet[2], John Robertson[3], Stéphane Mangin*[2], Weisheng Zhao*[1], and Tianxiao Nie*[1]

$These authors contributed equally
*These authors jointly supervised this work

This PDF file includes:

Sections S1 to S8
Figs. S1 to S15
Table S1
References

1. Sample characterizations

As shown in Fig. S1a, in Fe_3GeTe_2 (space group $P6_3/mmc$), Fe-Fe dumbbells form a triangular lattice that interpenetrates to the Fe-Ge honeycomb lattice. While in Fe_4GeTe_2 (space group R-3m), Fe-Fe dumbbells form a corrugated honeycomb lattice hosting one Ge atom at the center. Therefore, Fe_4GeTe_2 is expected to present a different crystal characterization from Fe_3GeTe_2 in XRD results. To demonstrate the structure difference clearly, Fe_4GeTe_2 and Fe_3GeTe_2 thin films with the same thickness were prepared and performed XRD measurements respectively. As shown in Fig. S1b, the Fe_3GeTe_2 shows the peaks belong to the {002} family, while only the {003} family peaks were obtained in Fe_4GeTe_2, indicating that no disorder and impurity phase is induced during the iron-enrichment epitaxy in this work. Fig. S2 shows the EDX spectrum of Fig. 1d. Three prominent peaks at 3.769 keV, 6.396 keV, and 9.874 keV were obtained, which correspond to the Te, Fe, and Ge elements, respectively. The quantitative analysis of EDX confirms the Fe concentration of 57% in our sample, which is consistent with the stichometry of Fe_4GeTe_2.

Fig. S3a shows the static MOKE results of the 4 nm Fe_4GeTe_2. Here the longitudinal (in-plane) and polar (out-of-plane) MOKE signals were measured by changing sample position and incident light. The clear magnetic hysteresis loops demonstrate the high-temperature magnetic order, and the steeper in-plane loop confirms the robust in-plane magnetic anisotropy (IMA) in the Fe_4GeTe_2. The high-temperature magnetism can also be verified by the anomalous Hall curves, as shown in Fig. S3b, in which S-shaped R-H hysteresis loops can be observed even at 350 K, indicating the high T_c in the Fe_4GeTe_2. Meanwhile, FMR measurements were performed further to verify the authenticity of the high-temperature T_c. A Vectorial Network Analyzer FMR setup was used to study the magnetic dynamics of the films, as shown in Fig. S3c. A series of in-plane field-dependent FMR curves were obtained under different resonance frequencies and a peak shift as the function of the field was observed, which is the typical ferromagnetic feature and proves the presence of room-temperature magnetism in our Fe_4GeTe_2. The magnetism can also be unveiled by the

in-plane M-T characterization under a high magnetic field of μ_0H = 1T in Fig. S3d, which shows a monotone decrease as the temperature increases. Here we extract a saturation magnetization (M_s) of 2.13 μ_B/Fe and the value is higher than that of Fe_3GeTe_2 (1.6 μ_B/Fe) single crystal[1].

Traditionally, either Fe_3GeTe_2 or Fe_4GeTe_2 suffers a dimensionality effect under the 2D scale, which results in a decrease of T_c with the thickness decrease. However, our Fe_4GeTe_2 samples show an anomalous T_c enhancement in the thinner film compared to its bulk counterpart due to the interfacial effect between the film and the substrate. To confirm its contribution, we prepared a mechanical exfoliated Fe_4GeTe_2 flake, which was cleaved from a 10 nm epitaxial Fe_4GeTe_2 film, as shown in Fig. S4a and S4b. The room-temperature Kerr curves of these two samples were performed by the Evico magnetics Kerr microscope system, as shown in Fig. S4c and S4d. After exfoliation from the substrate, the Fe_4GeTe_2 flake cannot sustain a room-temperature magnetism, proving the enhancement of T_c comes from the interfacial effect. This result is consistent with our theoretical calculation, which will be discussed later.

2. Electronic structures of Fe_4GeTe_2

The geometry optimized lattice constant for ML is a=b=4.08 Å, for bulk a=b=4.01 Å, c=32.79 Å. Fig. S5 shows the spin-resolved band structure and partial density of states (PDOS) of ML Fe_4GeTe_2. The blue lines in Fig. S5a are the majority spin bands that are occupied by the *d* electrons of Fe while the minority *d* bands are mostly unoccupied. Fig. S5b shows the PDOS for the single-layer Fe_4GeTe_2, the valence band DOS is dominated by the PDOS of Fe *d* orbital electrons, and the localized states (E_d) that determine the magnetic moments can be defined as the energy level of unpaired Fe *d* electrons. For α-Fe, the *d* localized states are deep level to -5 eV with higher spins, which is expected to provide more magnetic moments than the *d* electrons of β-Fe. For bulk Fe_4GeTe_2, the PDOS should be similar. The flat bands in Fig. S5a correspond to the Fe *d* bands.

3. Calculation of the exchange interactions

A straightforward way to extract the exchange interactions is by calculating the total energies of various mutual-orthogonal magnetic configurations and solving a matrix equation. However, the magnetic moment depends on the magnetic configurations in the iron-based compound. The calculated total energies consist of not only the Heisenberg part but also the magnetization energies. Here, we neglect the non-isotropic part of the matrix J_{ij} in the main text of Eq (2), which has been proved to be validated in previous research about Fe_3GeTe_2[2,3]. Another assumption we made is that the $J(r)$ dispersion should follow the RKKY rule in Eq (1), as shown in Fig. S6a. A more accurate routine for the treatment of the exchange interactions for itinerant magnetism is to deduce the exchange coefficients for each wave vector on the Fermi surface via adopting linear response theory combined with the magnetic force theorem. However, the approaches based on this routine are quite computational demanding[4,5]. In this work, we simplify the calculations by assuming a spherical Fermi surface with a single Fermi vector. This vector is estimated by averaging along the 2D Fermi surface and is able to manifest the itinerant magnetism of vdW magnets[6].

As shown in Fig. 2b, the exchange interactions are classified by the spatial distance. By drawing a circle around a certain site in the hexagonal Fe network, the number of identical Fe-Fe exchanges on the circle can be confirmed. Due to the dumbbell network in Fe_4GeTe_2, each point in Fig. 2b has two irons. J_i means the exchange between the center iron and irons on the ith circle, also one is the α-iron and the other should be β-iron. Here $J_{i\alpha}$ means the exchange between two α-irons, $J_{i\beta}$ means the exchange between two β-irons. Table. S1 shows the number of equivalent exchanges in each type.

To derive J in Eq (1), we calculate the energy difference between the ferromagnetic (FM) configuration and antiferromagnetic (AFM) configuration. As stated above, the magnitude of the magnetic moments in AFM configuration should equal that in FM configuration for both α-irons and β-iron to exclude the magnetization energies. The selected AFM configuration is FM in its sub-cell therefore the magnetic moments are preserved, as shown in Fig. S6b. On the other hand, if the sub-cell is set

to be AFM, the β-iron shall have no spin. The energy difference contains the exchanges from J_2 while J_1 is canceled out. Since $J_2/J_{2\beta}$ is in the same order as J_1, this method is good enough to capture the most important exchanges. Then the calculated k_F of both ML and bulk Fe_4GeTe_2 are 0.5 $Å^{-1}$, by evaluating the Fermi surface in 2D and 3D. Noted that k_F varies by applying strain. Lastly, with the J, we can plot the $J(r)$ dispersion, Fig. 2c for the bulk/heterostructure, and Fig. S6a for ML. In bulk Fe_4GeTe_2, there are interlayer exchanges apart from intralayer exchanges. However, the nearest interlayer Fe-Fe distance is 7.5 Å, which contributes little to the overall exchange interaction.

4. Monte Carlo calculation

With the single-ion anisotropy (SIA), the two-dimensional Heisenberg model should have FM order at a finite temperature[7]. Note that most previous theoretic analysis of the magnetic moment-temperature (M-T) relation is based on the Ising model, which is the limit of $A\rightarrow-\infty$ in Eq (2). We found Fe_4GeTe_2 is more like the Heisenberg type rather than the Ising type as SIA is weak compared to the largest exchange interaction. The critical exponent here is estimated to be β=0.34 (shown in both Fig. 1f and Fig. 2d), while β in Ising type 2D ferromagnets is ~1/8. Consequently, the Ising model overestimates T_c by constraining the spin degree of freedom to the easy axis. Here, we also consider the magnetic anisotropy transition at roughly 150 K, above which the shape anisotropy is dominant. The preferred direction is no longer an axis but a plane and inside the plane it is isotropic, corresponding to A>0. Noted that under the limitation of $A\rightarrow+\infty$, it corresponds to the XY model. The magnetic anisotropy depends on spontaneous magnetization[8]. The magnetocrystalline anisotropy which favors an easy-axis anisotropy is related to the magnetization by $\sim M^3$, while the shape anisotropy which favors the planar anisotropy is $\sim M^2$. A self-consistent calculation is needed to obtain the M-T curve and A-T curve simultaneously, which is tedious. Therefore, in accordance with the experimental results in Fig. 3c, we simplify the transition of SIA to be linear inside the transition region while constant outside, as

shown in Fig. 2d. Given that A is usually two orders less than J, the exchange interaction plays the main role in determining T_c.

5. Strain effect

The strain may come from the lattice mismatch between the Fe_4GeTe_2 film and the sapphire substrate, therefore its effect on the first few layers is strong but weakens as the film thickness increase. First, we investigate the strain effect of the ML Fe_4GeTe_2. The magnetic moment varies monotonically with the strain (Fig. S7a). As the lattice is squeezed, more electrons are forced to pair thereby the magnetic moment diminishes and vice versa. The spatial distances between Fe sites do not change much by strain, indicating that strain affects J^2, not the r, as shown in Fig. S7b. Notably, the minimum of J^2 is somewhere near 0%, which means that the J^2 will increase under either a compressive or tensile strain. However, the elastic energy will boost rapidly with a strain of more than 4%, as shown in Fig. S7c. Therefore, we infer the strain should be less than 4% in the interface. Considering the relatively high lattice mismatch between the Fe_4GeTe_2 film and the sapphire substrate, we proposed that the Fe_4GeTe_2 film follows a special epitaxial mode that can suppress the tensile strain while keeping its original hexagonal crystal structure, which will be discussed in the next part. Therefore, the contribution of the strain can be quantified by analyzing the DOS shift. Fig. S7d demonstrated that the valence band DOS will move towards the E_F by compression and away from the E_F by extension. This can help us to decouple the effect of the strain.

6. The interface of Fe_4GeTe_2/α-Al_2O_3

To explore the interface effect between the Fe_4GeTe_2 and the substrate (α-Al_2O_3), the lattice match scheme of Fe_4GeTe_2 must be figured out. Considering the relatively high lattice mismatch (~20%), a special epitaxial mode Fe_4GeTe_2:Al_2O_3=2:$\sqrt{3}$ was proposed as shown in Fig. S8a. By rotating 30º, the lattice mismatch of the Fe_4GeTe_2|Al_2O_3 heterostructure is suppressed to 2%. Density functional calculation reveals that the interfacial layer distance is 2.7Å. Fig. S8b shows the top view of the

interface under this lattice match scheme. After rotating 30º, the Fe_4GeTe_2 crystal demonstrates the same in-plane orientation as the Al_2O_3 crystal, as shown in the black arrow. For crystals with a hexagonal structure, the RHEED result may demonstrate a pattern along either [11-20] or [1-100] direction, according to the incident direction of the electron beam. The key feature to distinguish these patterns is the distance between the streaky lines (*D*), which was determined by the interplanar spacing (or *d*-spacing, *d*) of the crystal structure along the direction, and they always follow a relation as $D{\sim}1/d$. Because the [11-20] direction has a larger *d*-spacing, more streaky lines can be observed in the RHEED pattern, while the [1-100] direction has fewer streaky lines. Therefore, the Fe_4GeTe_2 in Fig. S8b is expected to present an RHEED feature of the same direction as the Al_2O_3 substrate, due to the same structural arrangement of the crystals. By contrast, if no rotation during the growth, the Fe_4GeTe_2 will keep a strict 1:1 epitaxial mode along with the structure of Al_2O_3. Under this growth mode, the RHEED of Fe_4GeTe_2 will demonstrate a different feature from that of Al_2O_3 because the angle between the crystal directions of the Fe_4GeTe_2 and the substrate is 30º, as shown in Fig. S8c. The epitaxial mode in Fig. S8b can be proved by the experimental results in Fig. S8d. After deposition, the RHEED of Fe_4GeTe_2 demonstrates the same line density as that of Al_2O_3 despite the different original RHEED directions, indicating the alignment of Al_2O_3 substrate and Fe_4GeTe_2 film, and proving the presence of crystal rotation of Fe_4GeTe_2 during the deposition.

The rotation epitaxial mode secures a relatively small mismatch as well as the strain during the Fe_4GeTe_2 growth, which is well consistent with our theoretical speculation (Fig. S7). The in-plane thickness-dependent lattice constants (*a*) of Fe_4GeTe_2 can be roughly extracted from the time-resolution RHEED pattern by $a_{Fe_4GeTe_2}:a_{Al_2O_3}=D_{Al_2O_3}:D_{Fe_4GeTe_2}$, which is shown in the inset of Fig. S8e. The initial Fe_4GeTe_2 suffers a trivial lattice extension and it shrinks quickly as the thickness increases, indicating the small lattice mismatch and strain during the growth.

7. Interface-induced electronic structure change in Fe_4GeTe_2

In Eq (3), we have proved that the J is related to the distance between the Fermi level (E_F) and the localized states (E_d). Considering the thickness dependency of the T_c in Fig. 1h, we assume that the shift of E_d induced by the interface is the reason for the T_c enhancement. Here we calculate the DOS of Fe_4GeTe_2 with and without interface. After integration with the sapphire, the valence band DOS moves towards E_F, as shown in Fig. S9a. Fig. S5b has indicated that the valence band of Fe_4GeTe_2 is dominated by Fe d bands, therefore, the DOS shift mainly comes from the redistribution of d orbital electrons in Fe_4GeTe_2, which indeed presents the same collapse towards E_F on a sapphire substrate (Fig. S9b). On the contrary, the PDOS of s and p orbitals are barely changed with an interface (Fig. S9c). Considering that α-Fe provides the largest magnetic moments, we calculate the PDOS of α-Fe d electrons to inspect the interface-induced localized states (E_d) shift, as shown in Fig. S9d. On the interface, the orbital coupling between Fe_4GeTe_2 and sapphire induces a right shift of Fe d PDOS, which will move the E_d towards the E_F, leading to an enhancement of J as well as the T_c. Notably, for Fe atoms on the surface side and the substrate side, their d orbitals PDOS suffer different delocalization, as the interfacial effect is more prominent on the substrate side, which is the reason for the thickness dependency in Fig. 1h.

To verify our theoretical calculation, we use the UPS spectrum to probe the E_d shift induced by the interface. The UPS measurements were carried out in a UHV vacuum chamber equipped with standard instruments for sample preparation. The valence band DOS for the sample was measured using monochromatized He-I radiation at 21.22 eV. All the photoemission spectra were recorded using a scienta SES-200 analyzer with an energy resolution better than 20 meV (pass energy 10). First, a 16 nm Fe_4GeTe_2 was deposited on a sapphire substrate followed by several times of ion etching (Ar+ with a beam energy of 800 eV) and UPS measurements. With etching, the film thickness can be decreased in situ and the contribution of the interface will be enhanced. Notably, although etching has been used as an applicable method to control the thickness of 2D materials[9,10], it is difficult to achieve it on an atomic scale. In this work, the removed thicknesses may be not so precise, which corresponds to the average

etching depth according to our previous calibration. Fig. S10 compares the valence band DOS we obtained by theoretical calculation and the experiments. After etching, a peak appears at ~4.1 eV, which corresponds to the Fe *d* localized states because of the PDOS shift. As the film thickness decreases, the peak becomes stronger due to the enhanced interfacial orbital coupling, as shown in Fig. S10b-d. The UPS spectrum is consistent with our calculated PDOS for α-Fe *d* electrons (Fig. S10a). A recent work[2] indicated that the T_c of the FGT can be enhanced by electron doping, which shifts the E_F and leads to a modulation in the magnetism as well as the carrier density. However, the contribution of the electron doping can be excluded because no E_F shift was observed in the zoom-in UPS spectrum (the inset of Fig. S10).

8. Stoichiometry-dependent magnetism in Fe_4GeTe_2

To explore the stoichiometry-dependent magnetism in Fe_4GeTe_2, three samples with the same thickness (10 nm) but different Fe concentrations were prepared by precise manipulation of the effusion rate during the growth. The samples were named $Fe_{4-x}GeTe_2$, Fe_4GeTe_2, and $Fe_{4+x}GeTe_2$, and the magnetic properties were characterized using a SQUID system. As shown in Fig. S11, the clear hysteresis loops can be observed in all the samples, even when the temperature reaches 300 K. Moreover, the results prove that a slight variation of Fe concentration can modulate the magnetic anisotropy in Fe_4GeTe_2. As shown in Fig. S11a, the $Fe_{4-x}GeTe_2$ demonstrates a robust PMA, by contrast, the $Fe_{4+x}GeTe_2$ film presents a strong IMA in all temperatures (Fig. S11c). This easy axis transition is the consequence of the effective magnetic anisotropy variation, which can be calculated as $K_{eff} = K_m + K_{sh}$. Here the K_m is magnetocrystalline anisotropy, which favors the out-of-plane direction, and K_{sh} is shape anisotropy which favors the in-plane direction[11]. They usually roughly follow a relation[12] as $K_{eff} = K_m + K_{sh} = K_m - (\mu_0 / 2)M_s^2$. Therefore, the competition between K_m and K_{sh} will tune the magnetic anisotropy in the samples. Notably, unlike the $Fe_{4+x}GeTe_2$ or $Fe_{4-x}GeTe_2$ where the easy axis was dominated by single anisotropy energy, the Fe_4GeTe_2 presents a temperature-dependent anisotropy (Fig. S11b). In low temperatures, the easy

axis lies in the *c* plane because of the considerable K_m contribution (K_{sh}=-0.4 J/cm^2 and K_m=0.88 J/cm^2 in 20 K). When the temperature increase, the K_m shrinks and K_{sh} start to dominate the K_{eff} (K_{sh}=-0.23 J/cm^2 and K_m=0.08 J/cm^2 in 300 K), resulting in a spin reorientation in Fe_4GeTe_2.

Besides the magnetic anisotropy, the T_c can also be modulated by stoichiometry. Fig. S12 demonstrates the out-of-plane high-temperature M-H loops of the three samples via VSM. The $Fe_{4-x}GeTe_2$ shows the lowest T_c of ~370 K, followed by the Fe_4GeTe_2 (T_c~430 K), and the $Fe_{4+x}GeTe_2$ has the highest T_c, which is almost 460 K. The accurate T_c summarized in Fig. 3b was obtained from the power-law fitting from the spontaneous magnetization, as shown in Fig. S13a-c, indicating the positive correlation between the Fe concentration and T_c. In addition, the Arrott plots can also be used to estimate the T_c of the ferromagnetic materials, as shown in Fig. S13d-f. And the T_c calculated from the Arrott plot is very close to that from power-law fitting, confirming that a magnetism enhancement can be induced by increasing the Fe concentration.

The high-temperature magnetism of the samples can be further demonstrated by other characterizations. Fig. S14 depicts the temperature-dependent M-H loops of 10 nm $Fe_{4+x}GeTe_2$ by MOKE. Consistent with the SQUID results in Fig. S11c, the $Fe_{4+x}GeTe_2$ demonstrates a strong IMA even at 400 K. In addition, because of the robust PMA in $Fe_{4-x}GeTe_2$, Hall measurements were performed to characterize its magnetism at high temperatures. Fig. S15a depicts the typical structure of the Hall bar device in this work. After applying a vertical magnetic field (H//*c*), a series of anomalous Hall effect (AHE) curves can be obtained to demonstrate the magnetism of $Fe_{4-x}GeTe_2$, as shown in Fig. S15b. A hysteresis loop with clear coercivity can exist even when the temperature is over 300 K, proving the presence of the high-T_c PMA of the $Fe_{4-x}GeTe_2$ sample in all temperatures. By contrast, the Fe_4GeTe_2 sample shows a temperature-dependent anisotropy, which has been discussed by AHE (Fig. 3g) and SQUID (Fig. S11b) measurements. This feature can also be verified by Hall measurement with an in-plane field (H//*ab*), and the results are shown in Fig. S15c. A square hysteresis loop

can be obtained at a low temperature because of the strong PMA. However, a degradation of the hysteresis can be observed after 150 K. Because the high-temperature magnetism of the film has already been proved by the anomalous Hall curves in Fig. 3g, the degradation is expected to come from the considerable contribution of IMA.

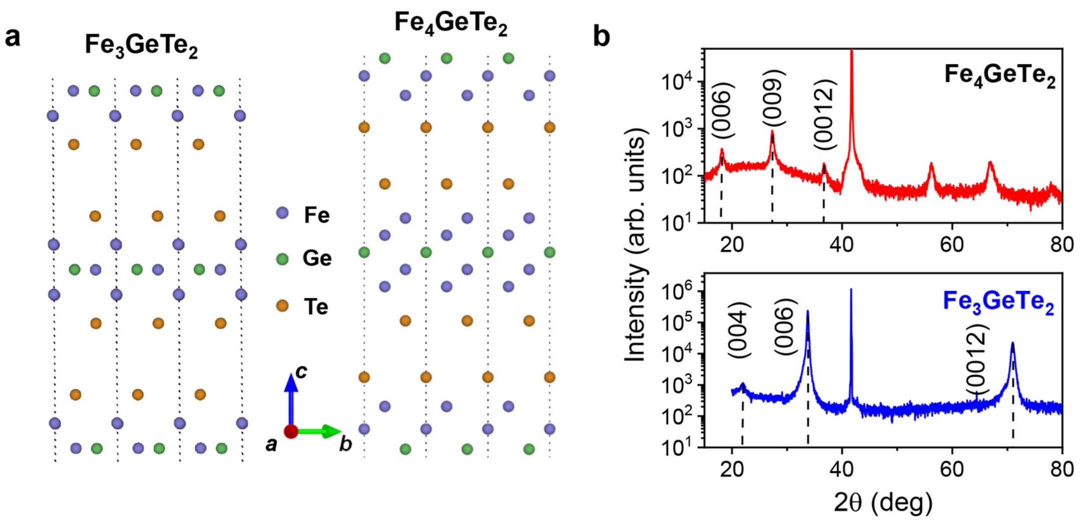

Fig. S1. The crystal structures of two Fe_nGeTe_2 vdW materials. (**a**) Two stable vdW structures in the series of Fe_nGeTe_2 identified for n = 3, 4. The structural units of Fe-Fe dumbbells form multiple-layer Fe-rich slabs, which are stacked by vdW coupling. The Fe_4GeTe_2 has a higher Fe concentration, which is expected to cause a different structural characterization with Fe_3GeTe_2 in XRD results in (**b**).

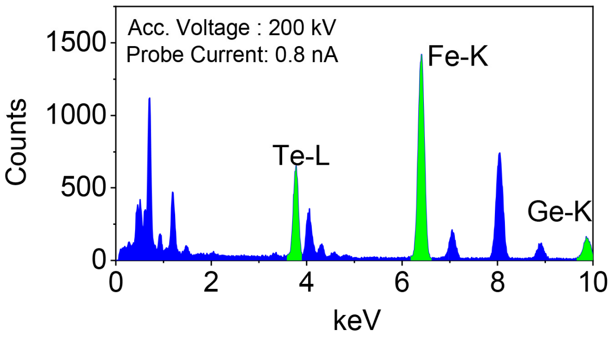

Fig. S2. The EDX result of our Fe_4GeTe_2 thin film. The stoichiometric composition is estimated as 4:1:2 Fe:Ge:Te.

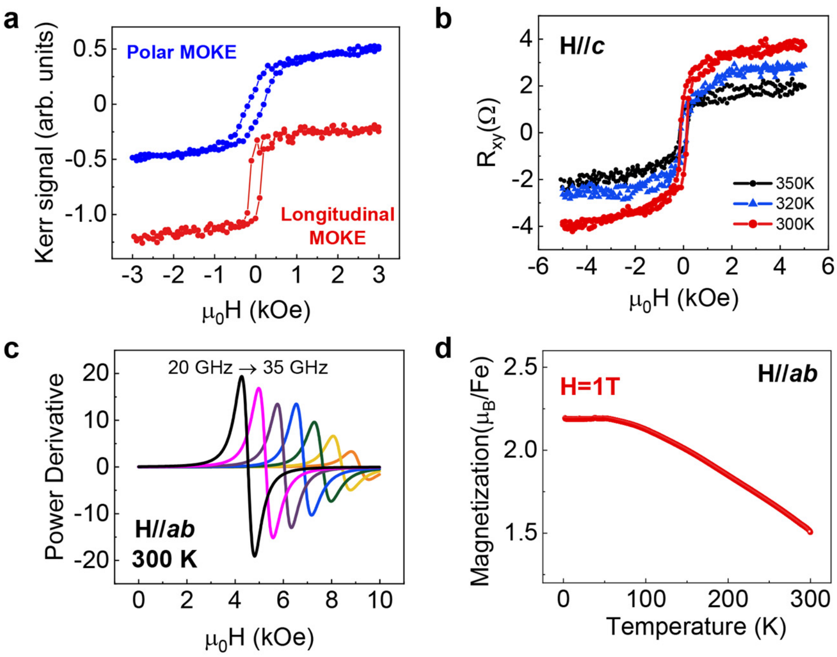

Fig. S3. Magnetic properties of Fe_4GeTe_2 thin film. (**a**) Polar (out-of-plane) and longitudinal (in-plane) MOKE for 4 nm Fe_4GeTe_2 at room temperature, the sample demonstrates an IMA feature. (**b**) Anomalous Hall curves for 7 nm Fe_4GeTe_2 at high temperatures. (**c**) The in-plane FMR profiles for 4 nm Fe_4GeTe_2, a peak shift with the magnetic field can be observed. (**d**) The in-plane M-T curve for 4 nm Fe_4GeTe_2 at H=1T.

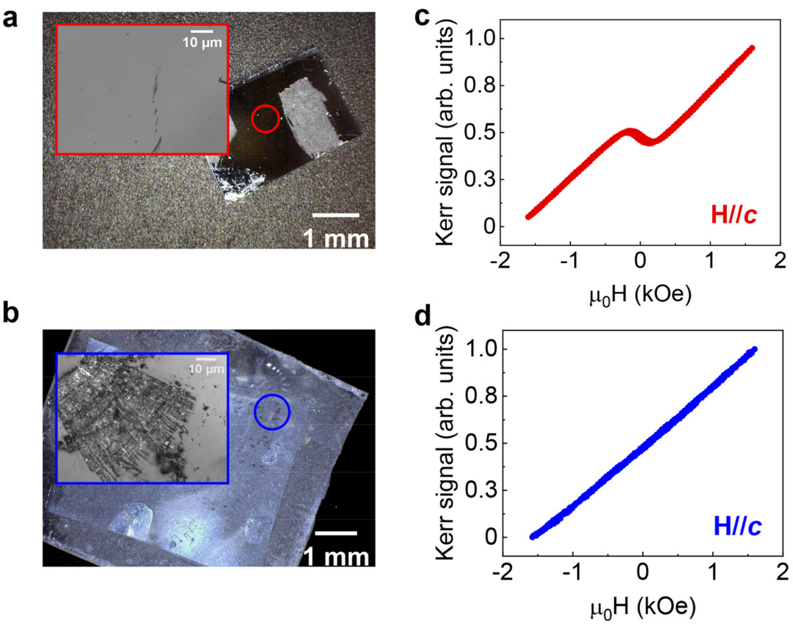

Fig. S4. Magnetic properties of the epitaxial and the exfoliated Fe_4GeTe_2 samples. (**a, b**) Optical micrographs of the 10 nm Fe_4GeTe_2 thin film and cleaved Fe_4GeTe_2 flake. After exfoliation, the Fe_4GeTe_2 flake was collected using adhesive tape and measured directly by the MOKE microscope. (**c, d**) Room-temperature MOKE curves of the samples in (**a**) and (**b**) for H//*c*. After exfoliation from the substrate, the enhanced magnetism disappears in the Fe_4GeTe_2 flake.

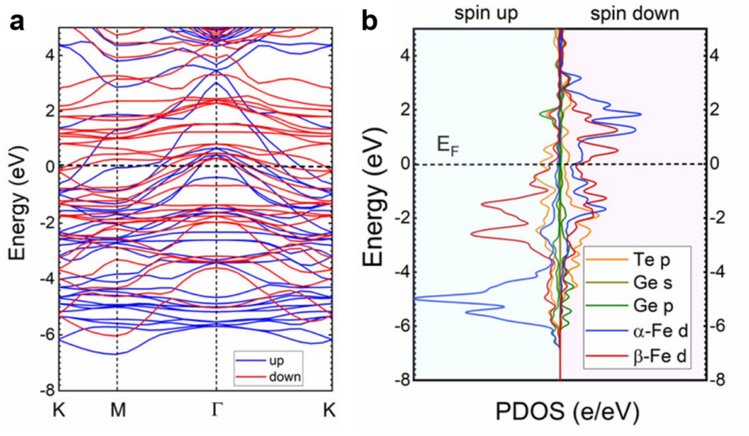

Fig. S5. Electronic structures of Fe_4GeTe_2. DFT calculated band structure (**a**) and PDOS by atomic species and orbitals (**b**) of ML Fe_4GeTe_2.

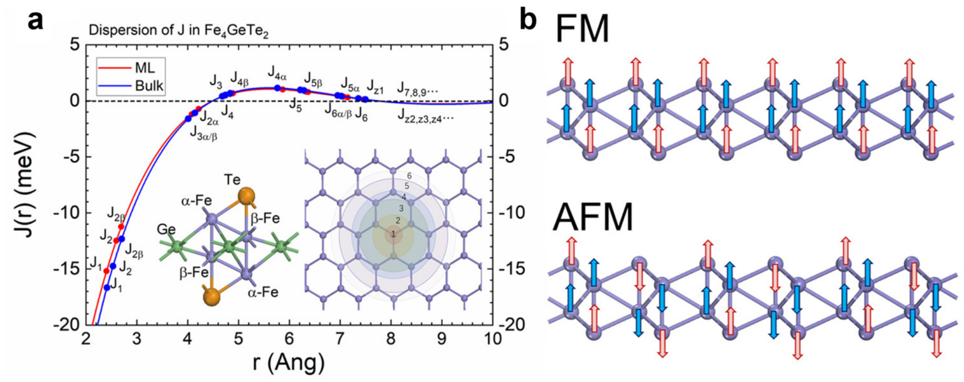

Fig. S6. Calculated exchange interactions of Fe_4GeTe_2. (**a**) *J*-*r* dispersion of monolayer and bulk Fe_4GeTe_2. Inset: atomic structure of monolayer Fe_4GeTe_2 with the naming rules. The exchanges couplings of ML and bulk are similar. (**b**) FM and AFM configurations used to calculate *J*. The magnetic moment $M_\alpha=3\mu_B$, corresponding to the red arrows; $M_\beta=2\mu_B$, corresponding to the blue arrows.

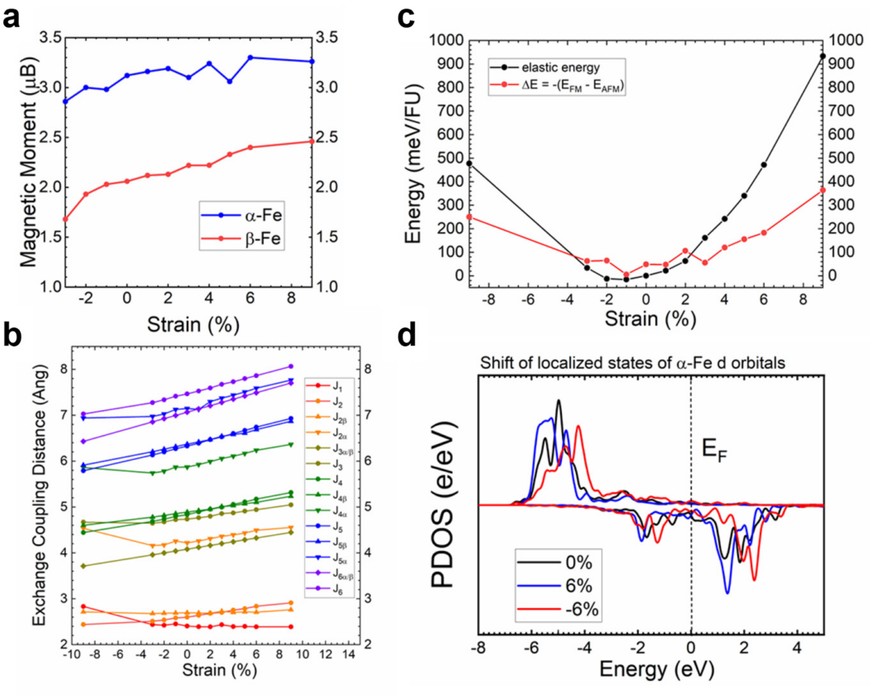

Fig. S7. Strain effect in ML Fe_4GeTe_2. (**a**) Strain dependence of the magnetic moment in α-Fe and β-Fe. (**b**) The Fe-Fe spatial distances versus strain from the nearest couplings to higher orders. (**c**) Strain elastic energy per formula unit (FU) and the energy difference between FM and AFM. (**d**) PDOS of relaxed, 6% extended and -6% compressed Fe_4GeTe_2.

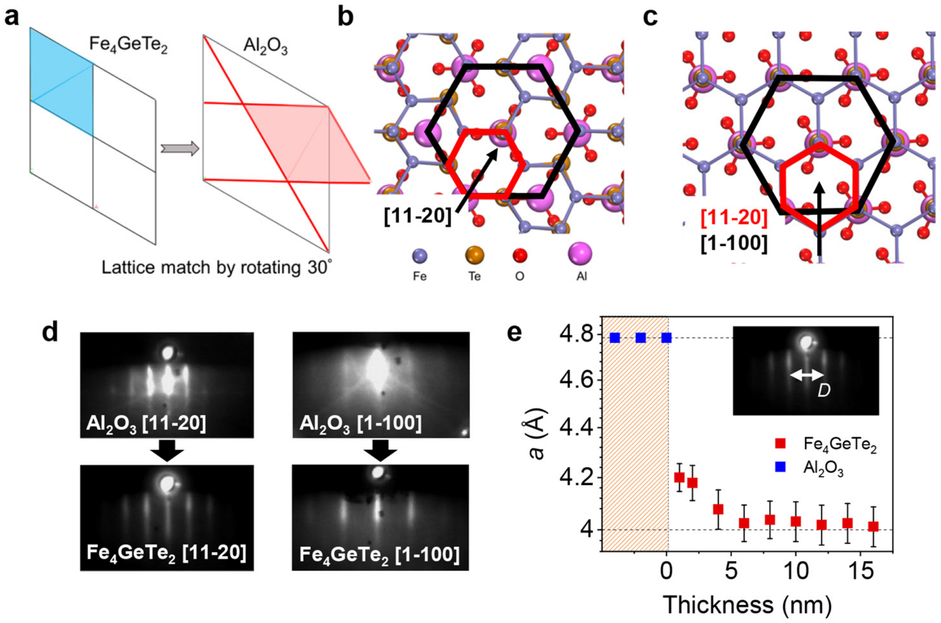

Fig. S8. The lattice match scheme between Fe_4GeTe_2 and Al_2O_3. (**a**) The proposed lattice match scheme. The blue region is the primitive cell of Fe_4GeTe_2, while the red region is the primitive cell of Al_2O_3. (**b**) Top view of the interface, with only the interfacial Fe, Te, Al, and O shown. After rotating 30°, the Fe_4GeTe_2 crystal (red) demonstrates the same in-plane orientation as the Al_2O_3 crystal (black) (**c**) 1:1 epitaxial mode of Fe_4GeTe_2, a 30° include angle between the Fe_4GeTe_2 crystal and Al_2O_3 crystal can be observed. (**d**) Observed RHEED patterns before and after Fe_4GeTe_2 deposition along with the different original directions of Al_2O_3 substrates, the Fe_4GeTe_2 perfectly inherits the RHEED features of Al_2O_3, indicating the same atomic alignment between these materials. (**e**) In-plane lattice constants as a function of thickness for Fe_4GeTe_2. Inset: a typical RHEED of Fe_4GeTe_2 along [11-20] direction.

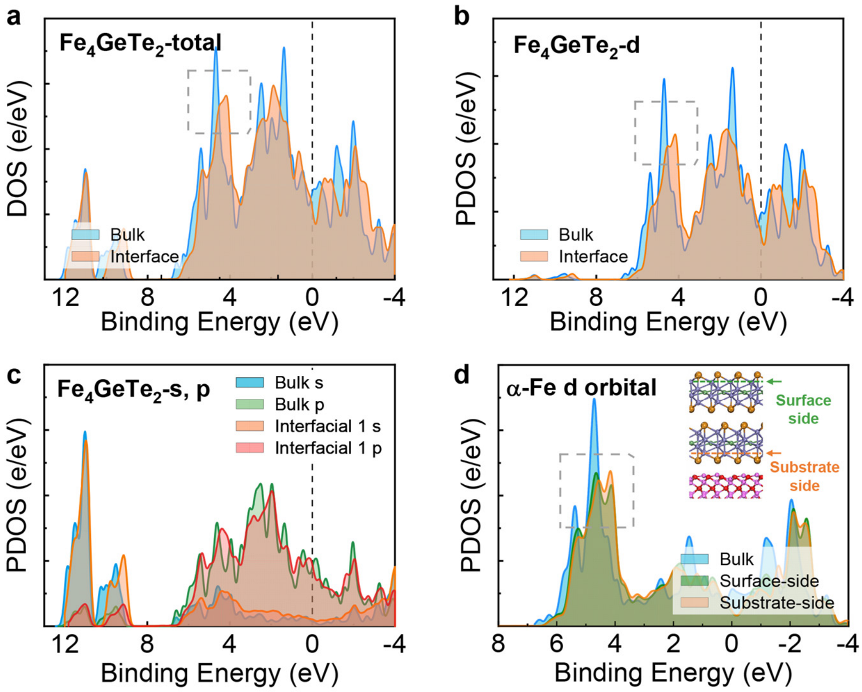

Fig. S9. The calculated (**a**) total DOS, (**b**) *d* orbital electrons PDOS, (**c**) *s* and *p* orbital electrons PDOS, and (**d**) α-Fe *d* orbital PDOS for Fe_4GeTe_2 with and without the interface. The dashed boxes indicate the DOS shift induced by the interface.

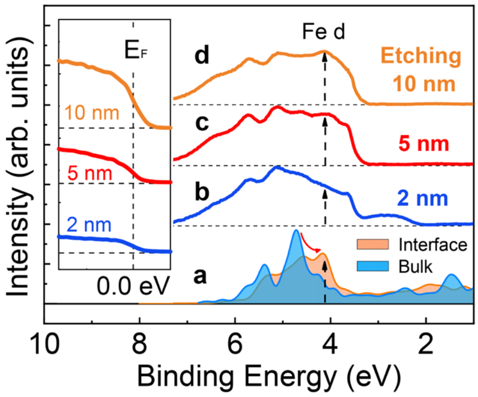

Fig. S10. The electronic structure change with film thickness (**a**) The calculated *d* orbital PDOS for the α-Fe in bulk and interfacial Fe_4GeTe_2. (**b, c, d**) The UPS spectrums for the valence band DOS after etching 2 nm, 5 nm, and 10 nm. Inset: the DOS near the E_F for **b**, **c**, and **d**.

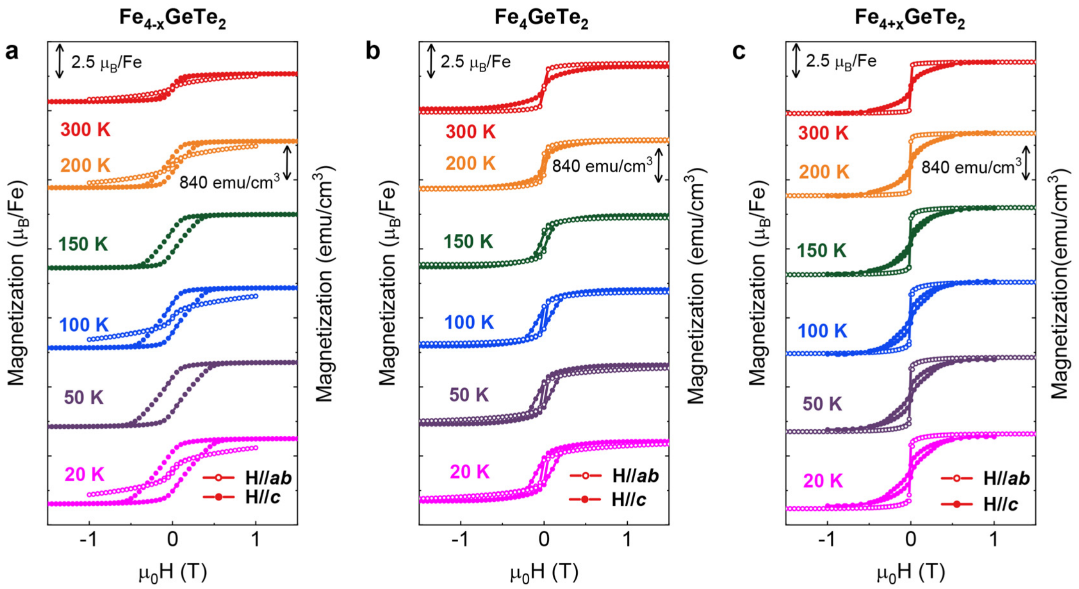

Fig. S11. M-H loops of the samples with different Fe concentrations. Out-of-plane and in-plane M-H loops from 10 nm (**a**) $Fe_{4-x}GeTe_2$, (**b**) Fe_4GeTe_2 and (**c**) $Fe_{4+x}GeTe_2$ at different temperatures. As the Fe concentration increases, the magnetic anisotropy turns from PMA to IMA. A temperature-dependent spin reorientation can be observed in Fe_4GeTe_2 in (**b**).

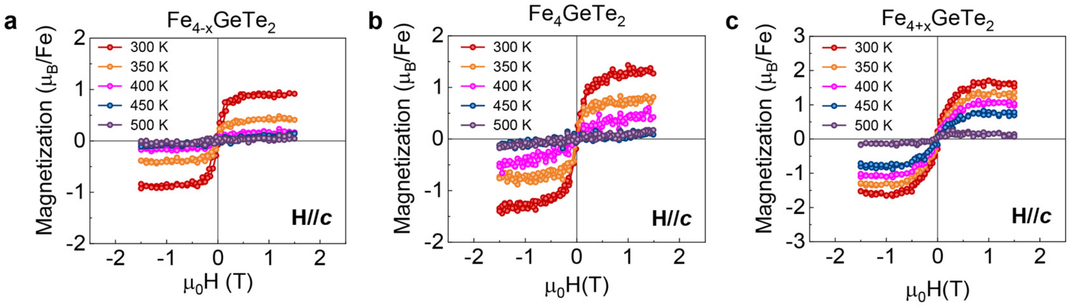

Fig. S12. High-temperature M-H loops of 10 nm (**a**) $Fe_{4-x}GeTe_2$, (**b**) Fe_4GeTe_2 and (**c**) $Fe_{4+x}GeTe_2$. All the samples show ferromagnetism above room temperature.

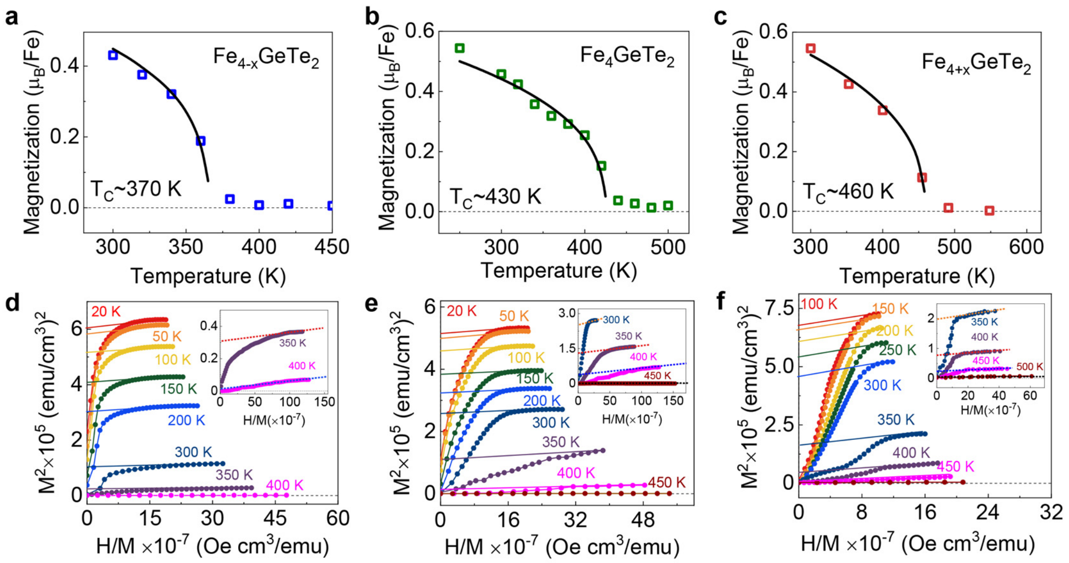

Fig. S13. Stoichiometry-dependent T_c of Fe_4GeTe_2 films with different Fe concentrations. (**a-c**) The power-law fitting curves of $Fe_{4-x}GeTe_2$, Fe_4GeTe_2 and $Fe_{4+x}GeTe_2$, and (**d-f**) the corresponding Arrott plots. The T_c increases with the increase of Fe concentration.

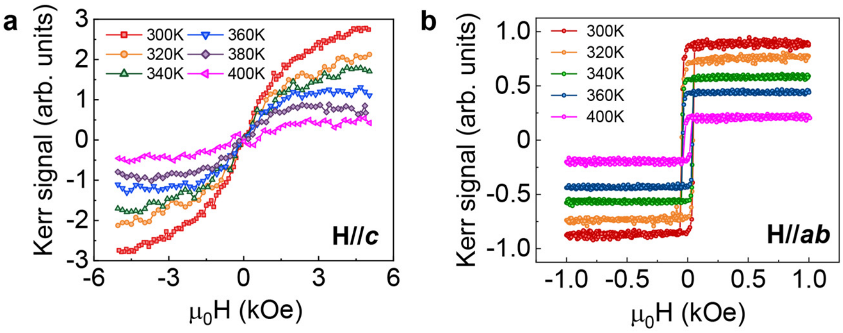

Fig. S14. High-temperature MOKE of 10 nm $Fe_{4+x}GeTe_2$ under (**a**) out-of-plane field and (**b**) in-plane field, the magnetism can survive even at 400 K.

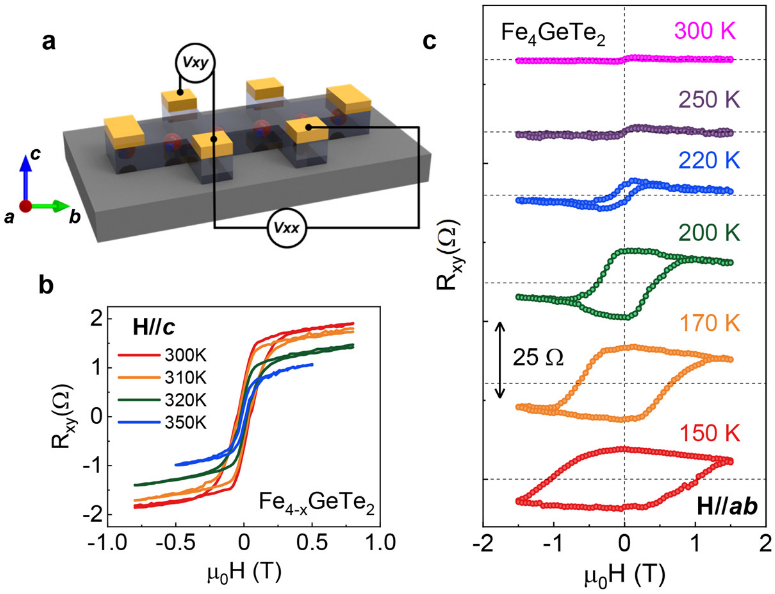

Fig. S15. Transport measurement. (**a**) Schematic of the Hall bar device. (**b**) High-temperature AHE curves for 7 nm $Fe_{4-x}GeTe_2$, the hysteresis loops with clear coercivities prove the high-T_c PMA of the sample. (**c**) In-plane Hall curves for 7 nm Fe_4GeTe_2, a degradation of the hysteresis loop can be observed, indicating the presence of anisotropy rotation from PMA to IMA with the temperature increase.

Table. S1. The number of equivalent exchanges in each type.

i	α-iron			β-irons		
	J_i	$J_{i\alpha}$	$J_{i\beta}$	J_i	$J_{i\alpha}$	$J_{i\beta}$
1	1	0	0	1	0	0
2	3	3	0	3	0	3
3	6	6	0	6	0	6
4	3	3	0	3	0	3
5	6	6	0	6	0	6
6	6	6	0	6	0	6

References

1. Chen, B. *et al.* Magnetic Properties of Layered Itinerant Electron Ferromagnet Fe_3GeTe_2. *J. Phys. Soc. Jpn.* **82**, 124711 (2013).
2. Deng, Y. *et al.* Gate-tunable room-temperature ferromagnetism in two-dimensional Fe_3GeTe_2. *Nature* **563**, 94-99 (2018).
3. Shen, Z.-X., Bo, X., Cao, K., Wan, X. & He, L. Magnetic ground state and electron-doping tuning of Curie temperature in Fe_3GeTe_2: First-principles studies. *Phys. Rev. B* **103**, 085102 (2021).
4. Wan, X., Yin, Q. & Savrasov, S. Y. Calculation of Magnetic Exchange Interactions in Mott-Hubbard Systems. *Phys. Rev. Lett.* **97**, 266403 (2006).
5. Zimmermann, B. *et al.* Comparison of first-principles methods to extract magnetic parameters in ultrathin films: Co/Pt(111). *Phys. Rev. B* **99**, 214426 (2019).
6. Seo, J. *et al.* Tunable high-temperature itinerant antiferromagnetism in a van der Waals magnet. *Nat. Commun.* **12**, 2844 (2021).
7. Mermin, N. D. & Wagner, H. Absence of Ferromagnetism or Antiferromagnetism in One- or Two-Dimensional Isotropic Heisenberg Models. *Phys. Rev. Lett.* **17**, 1133-1136 (1966).
8. Seo, J. *et al.* Nearly room temperature ferromagnetism in a magnetic metal-rich van der Waals metal. *Sci. Adv.* **6**, eaay8912 (2020).
9. Park, H., Shin, G. H., Lee, K. J. & Choi, S.-Y. Atomic-scale etching of hexagonal boron nitride for device integration based on two-dimensional materials. *Nanoscale* **10**, 15205-15212 (2018).
10. Jia, J. *et al.* Plasma-Treated Thickness-Controlled Two-Dimensional Black Phosphorus and Its Electronic Transport Properties. *ACS Nano* **9**, 8729-8736 (2015).
11. Laan, G. v. d. Microscopic origin of magnetocrystalline anisotropy in transition metal thin films. *J. Phys.: Condens. Matter* **10**, 3239-3253 (1998).
12. Ribeiro, M. *et al.* Large-scale epitaxy of two-dimensional van der Waals room-temperature ferromagnet Fe_5GeTe_2. *npj 2D Materials and Applications* **6**, 10 (2022).
